# A pathophysiological and mechanistic review of chronic inflammatory demyelinating polyradiculoneuropathy therapy

**DOI:** 10.3389/fimmu.2025.1575464

**Published:** 2025-04-14

**Authors:** Marta Caballero-Ávila, Lorena Martin-Aguilar, Roger Collet-Vidiella, Luis Querol, Elba Pascual-Goñi

**Affiliations:** ^1^ Neuromuscular Diseases Unit, Department of Neurology, Hospital de la Santa Creu i Sant Pau, Institut d’Investigació Biomèdica Sant Pau, Universitat Autònoma de Barcelona, Barcelona, Spain; ^2^ Neuromuscular Diseases, Centro para la Investigación Biomédica en Red en Enfermedades Raras (CIBERER), Madrid, Spain

**Keywords:** chronic inflammatory demyelinating polyradiculoneuropathy, complement, anti-FcRn, pathophysiology, treatment

## Abstract

Chronic inflammatory demyelinating polyradiculoneuropathy (CIDP) is an immune-mediated disease of the peripheral nerves characterized by proximal and distal muscle weakness and sensory abnormalities. CIDP has been associated with various pathophysiological mechanisms that are not fully understood and that likely differ across groups of patients. It has been proposed that an interplay of different immunopathological mechanisms including the cellular, humoral and complement pathways play a key role in peripheral nerve damage in CIDP. Currently approved treatments and therapies in research often target different potential pathophysiological mechanisms. The efficacy of these different treatments can shed light on the prominence of particular pathophysiological pathways in subsets of patients with CIDP. For example, the complement pathway plays a key role in promoting macrophage-mediated demyelination, and complement inhibitors are under development as new targets in CIDP treatment, with mixed results. The neonatal Fc receptor (FcRn) has also been targeted as a promising treatment avenue due to its role in immunoglobulin G degradation. Efgartigimod is the first FcRn blocker approved for the treatment of CIDP. This review provides an overview of key proposed mechanisms of action in CIDP pathophysiology in the context of both basic scientific findings and treatment targets in recent clinical studies.

## 1 Introduction

### 1.1 CIDP background

Chronic inflammatory demyelinating polyradiculoneuropathy (CIDP) is an immune-mediated syndrome (1) characterized by a progressive or relapsing–remitting course that progresses for more than eight weeks, and typically results in proximal and distal weakness and sensory loss (2, 3). Although pathophysiology may differ across groups of patients, it is widely accepted that these deficits arise as a result of an autoimmune attack to the peripheral nerves, which damages the myelin sheath (demyelination) of motor and sensory nerves. This damage leads to a reduction in conduction velocity and conduction blocks at the motor nerve fibers, and subsequent weakness and sensory loss.

CIDP is the most common chronic autoimmune peripheral nervous system disorder, with a prevalence that varies between studies and different populations. A recent systematic review of literature reported the prevalence ranged between 0.67 and 10.3 per 100,000 (4). This difference in prevalence is likely due to global variations in the diagnostic criteria (5, 6). Overall, CIDP is also reported to be more common in males and in people over 50 years of age (4).

### 1.2 CIDP diagnosis

Numerous sets of diagnostic criteria exist to diagnose CIDP. CIDP diagnosis is based on clinical, electrodiagnostic, and supportive information, according to the European Academy of Neurology/Peripheral Nerve Society (EAN/PNS) 2021 guidelines for the diagnosis and treatment of CIDP (7). Patients with suspected CIDP are classified into two diagnostic certainty levels, CIDP or possible CIDP (7). Different disease variants are now specifically defined by the diagnostic guidelines. Electrodiagnostic criteria are based on the presence of demyelinating features in nerve conduction studies. Supportive criteria, including imaging studies, cerebrospinal fluid protein content, nerve biopsy and response to treatment support the diagnosis of CIDP, when clinical and electrodiagnostic criteria allow only a diagnosis of possible CIDP. Despite the exhaustive diagnostic criteria, misdiagnosis of CIDP is very frequent, particularly for CIDP variants. Since a correct diagnosis is crucial for initiating effective and appropriate treatment and management of the condition, misdiagnosis can lead to a significant burden for patients and the healthcare system (6, 8, 9).

### 1.3 CIDP pathophysiology

CIDP is a syndrome formulated based on clinical criteria that do not reflect its immunopathological diversity. CIDP has been associated with various pathophysiologic mechanisms that are not fully understood and that likely differ across groups of patients. It has been proposed that an interplay of different immunopathological mechanisms including the cellular, humoral and complement pathways play a key role in peripheral nerve damage in CIDP (1, 10). The extent to which each of these mechanisms is active, in each disease variant within the CIDP spectrum, is unknown. Moreover, patients with the same CIDP variant can exhibit varying responses to treatments. This pathophysiological diversity is likely responsible for the different responses to different treatments.

Evidence for involvement of cellular immunity can be found in CIDP pathology and is characterized by T-cell and macrophage infiltration in peripheral nerves and nerve roots (11).

T-cell activation and subsequent expression of pro-inflammatory cytokines, and the presence of CD4+ and CD8+ T cells infiltrating sural nerve biopsies, suggests an important role for T cells in CIDP, while macrophage infiltration of the nerves triggers myelin breakdown through phagocytosis (12–14).

The exact role of B cells in CIDP pathogenesis is unknown; however, it has been reported that B cell phenotypes are altered in CIDP. Evidence for involvement of humoral immunity in CIDP includes the deposition of immunoglobulin G and M (IgG and IgM, respectively) on the surface of Schwann cells and the compact myelin in the peripheral nerves of patients with CIDP (15). Antibodies against different proteins of the node of Ranvier (contactin-1, contactin-1 associated protein 1, neurofascin 155 and nodal isoforms of neurofascin) were initially described in patients fulfilling the CIDP diagnostic criteria (16, 17). The distinct clinical features, immunopathology and response to therapy of neuropathies mediated by these antibodies, that are primarily of the IgG4 isotype, led to the creation of the new diagnostic category of “autoimmune nodopathies” in the updated EAN/PNS guideline, that is now considered a separate disease from CIDP (7). In CIDP, it is believed that autoantibodies specific to peripheral nerve antigens (that have not yet been identified) may drive macrophage phagocytosis through immunoglobulin (IG) Fc receptors, or via activation of the complement system.

**Figure 1:**
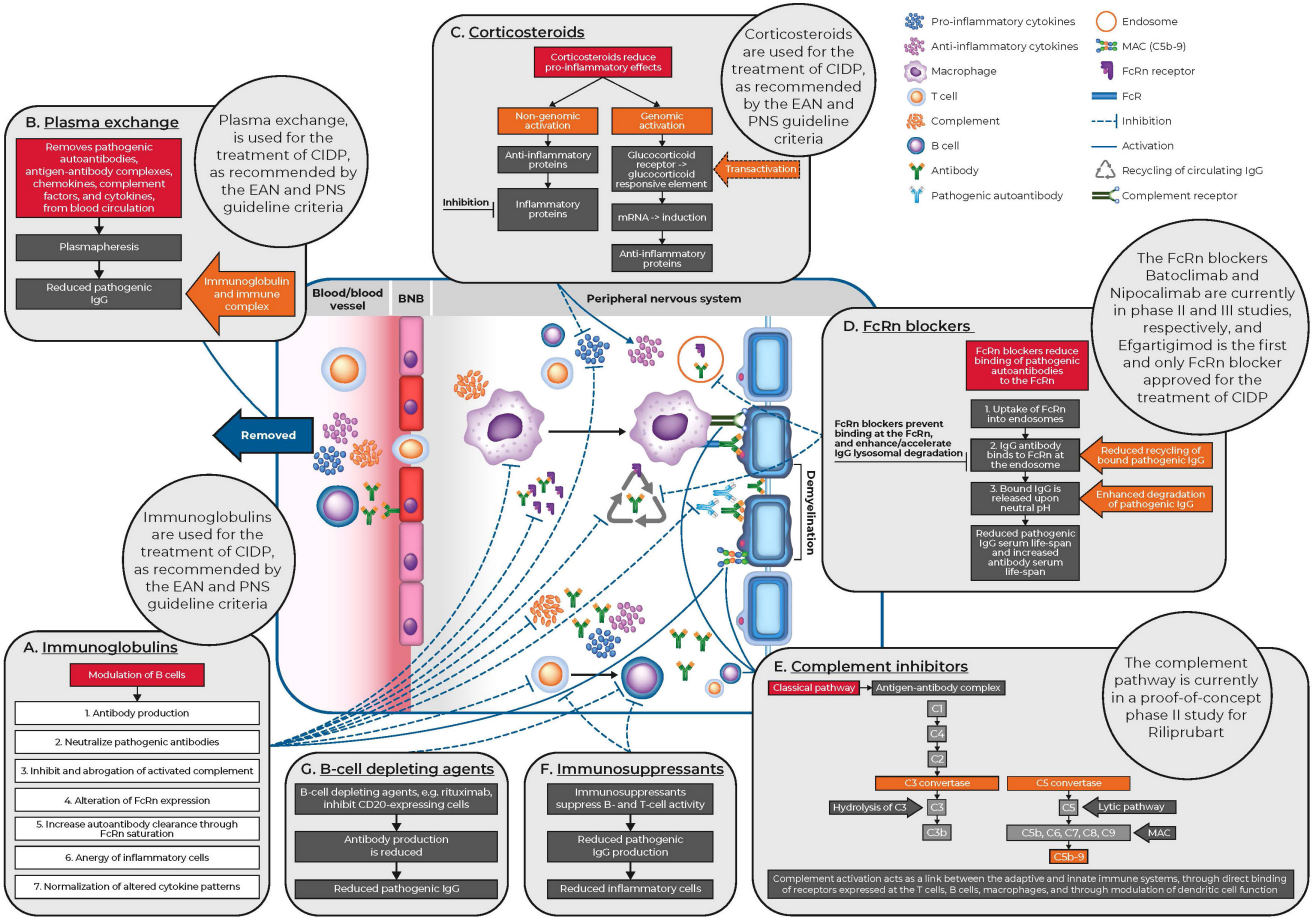
CIDP pathophysiology and mechanisms of action of current and novel treatments for CIDP. BNB, blood nerve barrier; CIDP, chronic inflammatory demyelinating polyneuropathy; EAN, European Academy of Neurology; FcR, fragment crystallizable portion receptors; FcRn, neonatal Fc receptor; IgG, immunoglobulin G; MAC, membrane attack complex; mRNA, messenger ribonucleic acid; PNS, Peripheral Nerve Society.

The complement pathway plays a key role in innate immune defense and tissue remodeling. Complement activation links the innate and adaptive immune systems by acting as the main effector mechanism of antigen-specific antibodies, by directly binding with receptors on T cells, B cells and macrophages, or by modulating the function of dendritic cells. The complement pathway consists of three independent pathways including classical (C1q), lectin (mannose-binding lectins or ficolin) and alternative (C3 autoactivation or properdin) (18) (**Figure 1**). The complement pathway has been targeted for its therapeutic potential. Complement capture and inhibition are among the mechanisms of action of IGs, which is an effective and widely used therapy in CIDP (10, 19–21).

In this narrative review, we will discuss both current and new treatment options and their mechanisms of action, for patients with CIDP. This article will not consider autoimmune nodopathies, as these are no longer identified as CIDP (7).

## 2 CIDP treatment

Since there are multiple phenotypic variations in clinical presentation and diverse pathophysiological mechanisms involved, treatment of patients with CIDP is complex and typically tailored to the individual patient. Selection of the most appropriate treatment typically involves a trial-and-error strategy as biomarkers required to identify which of the several mechanisms of disease within CIDP are predominant in a particular patient are lacking. Current standard of care (SoC) includes intravenous or subcutaneous IG (IVIG or SCIG), corticosteroids or plasma exchange (22, 23). These treatments are typically used as induction therapies (except SCIG) or as maintenance treatment in patients who require it (9, 14).

**Figure 2:**
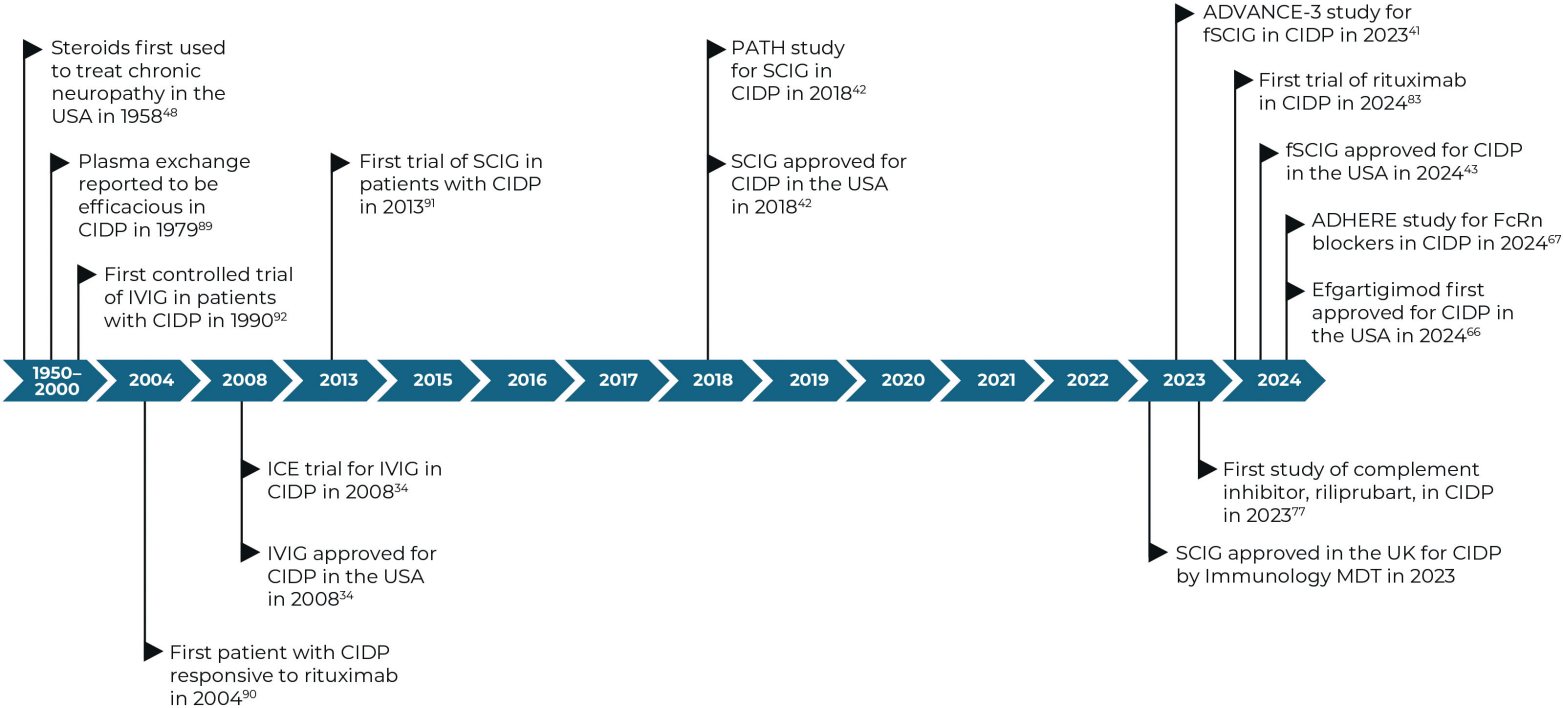
Timeline of key dates for treatments for CIDP and their approvals (28–40). CIDP, chronic inflammatory demyelinating polyneuropathy; FcRn, neonatal Fc receptor; IVIG, intravenous immunoglobulin; MDT, multi-disciplinary team; SCIG, subcutaneous immunoglobulin.

However, not all patients with CIDP respond to existing SoC treatments and approximately 10–25% of patients show resistance to all SoC treatments (24, 25); historically only 11% of patients achieved long-term remission or a cure over 5 years (26), and a 2022 meta-analysis reported a pooled remission rate of 40.8% from six studies (27). The heterogeneous nature of the disease pathophysiology may underpin the variation in responses to current SoC treatments as the predominant mechanism of disease may vary from patient to patient. Importantly, since misdiagnosis is frequent in CIDP, re-evaluation of the diagnosis is advised before escalating treatment in patients who do not respond to first-line treatments. This has opened other treatment avenues to help eradicate and target refractoriness to first-line therapies, residual disability, side effects to available treatment and cost and availability, particularly when considering long-term treatment options. **Figure 2** outlines the treatments of CIDP over the years, including treatments of unproven efficacy and those under current investigation.

### 2.1 Immunoglobulin treatment

IGs or antibodies are glycoproteins produced by B cells and plasma cells in response to a variety of antigenic stimuli. These medicinal products are purified from human plasma, obtained via donation.

The mechanism of action of IGs in CIDP treatment is both complex and multiple. IVIG treatment for patients with CIDP, acts through multiple mechanisms of action including neutralization of pathogenic autoantibodies, inhibition and abrogation of activated complement, alteration of Fc receptor expression, increased autoantibody clearance through FcRn saturation, anergy of inflammatory cells, and normalization of altered cytokine patterns (3, 41–44) (**Figure 1**). The most common IVIG induction and maintenance doses in clinical trials are 2 and 1 g/kg every three weeks, respectively, but maintenance doses vary in real-world clinical practice (45, 46). Common adverse effects following IVIG therapy include headaches, pyrexia, and hypertension and are usually mild in severity (28, 47).

Five randomized controlled trials have all demonstrated beneficial, yet short-term IVIG efficacy vs. placebo between 1993 and 2008, with 37–75% of patients demonstrating measurable improvement of their physical symptoms (28, 48–51). A 2017 open-label phase III trial of IVIG demonstrated long-term efficacy, with approximately 70% of patients having sustained remission for 52 weeks (52). Major barriers to IVIG use include the high cost, availability and inconvenience to patients due to administration at a hospital or daycare facility. Logistically less complicated than IVIG, SCIG provides an alternative treatment option that can be self-administered at home, allowing for more flexibility and autonomy. Compared with patients dependent on IVIG treatment or receiving placebo, evidence suggests that SCIG is safer and more effective as a maintenance treatment for CIDP (29, 30). Mild, local infusion-site reactions are reported as the most common adverse event among patients receiving SCIG, as well as headaches and fatigue (30). See **Supplementary Table 1** for a summary of IG clinical trials in CIDP.

Utilization of IVIG and SCIG for the treatment of CIDP has been around for 3 decades. Based on response rates from clinical trials and real-world use, most patients may benefit from this treatment, as it targets multiple mechanisms of disease of CIDP. The targeting of IGs and their pathways for the treatment of CIDP is continuously developing, with facilitated SCIG (fSCIG) demonstrating a similar efficacy to IVIG and recently being approved for the treatment of CIDP (29, 31) (**Figure 2**).

### 2.2 Plasma exchange

Plasma exchange, also referred to as plasmapheresis, is a technique that replaces plasma in the blood of patients. The procedure removes substances of high molecular weight such as antibodies, antigen-antibody complexes, cytokines, chemokines and complement factors (53) (**Figure 1**). It has been demonstrated that the sera from patients with CIDP can cause demyelination or functional peripheral nerve deficits following intraneural or systemic transfer in animal models, providing evidence for the use of plasma exchange in CIDP (54). Two randomized controlled trials compared plasma exchange with sham exchange. Overall, both trials reported that plasma exchange indicated improved short-term outcomes, while the second study by Hahn et al., reported subsequent re-deterioration within eight weeks (55). This suggests that plasma exchange can be used in the acute disease phase, especially in severely affected patients, while other treatments are required for long-term therapy (55, 56). Reported adverse effects of plasma exchange for CIDP include hypotension and citrate reactions (56).

Patient responses to plasma exchange indicate that humoral factors (autoantibodies, cytokines, chemokines and complement) may all be involved in CIDP pathophysiology; however, the administration logistics of plasma exchange mean that other treatments are required.

### 2.3 Corticosteroid treatment

For decades, natural and synthetic corticosteroids have been among the most prescribed class of drug for immunomodulation. Corticosteroids were first described as a treatment option for patients with recurrent polyneuropathies in 1958 (32) (**Figure 2**), and similar to IVIG treatment, can be offered as a first-line treatment to newly diagnosed patients with moderate or severe disability (57). Patients should be carefully monitored for treatment response, which usually starts after several weeks or months of initiating treatment (7). Corticosteroids are anti-inflammatory and immunosuppressive, and mediate genomic effects that increase the production of anti-inflammatory proteins and reduce the production of pro-inflammatory proteins (58). Corticosteroids also have non-genomic effects through heterogeneous receptors and pathways with similarly complex impacts (57, 59) (**Figure 1**). Importantly, corticosteroid-mediated apoptosis of multiple cell types of hematopoietic origin, and suppression of pro-inflammatory cytokine gene expression are the key primary mechanisms of action that lead to anti-inflammatory responses (60). As lipid-soluble anti-inflammatory agents, corticosteroids can easily cross the cell membrane and bind to the glucocorticoid receptor. This receptor complex can modulate the expression of various genes, resulting in a pleiotropic anti-inflammatory effect mainly related to cytokine modulation and facilitation of T-cell apoptosis directed against peripheral nerves (57).

Several studies have shown that corticosteroids display similar benefits to IVIG in patients with CIDP, however, corticosteroid treatment may provide longer therapy-free remission or increased remission rates when compared with IVIG (61, 62). The PREDICT study compared daily oral prednisolone with monthly pulse oral dexamethasone (63). The study of 41 participants showed no difference in the primary outcomes and patients achieving remission without treatment in 12 months. Reported adverse events were mostly mild; sleeplessness and Cushing’s face occurred most often in patients who received prednisolone (63).

However, this study supported the use of pulse therapy, which provided a faster speed of action and fewer side effects than long-term prednisolone schedules. A retrospective study evaluated three treatment regimens (daily oral prednisolone, pulsed oral dexamethasone, and pulsed intravenous methylprednisolone) in patients with CIDP. Overall, corticosteroid treatment resulted in an improvement in 60% of patients and achieved remission in 61% of those who responded to treatment, with no notable differences in safety or effectiveness among the regimens (64).

Recently, the OPTIC study investigated the combination of IVIG and corticosteroids in patients with CIDP, with the hypothesis that the combination would lead to more frequent long-term remission compared with IVIG alone (65). Unfortunately, this study had to be stopped prematurely for safety reasons as four thromboembolic events were detected in the combination group; results from this trial have been presented at the PNS 2024 annual meeting and are currently awaiting publication (66). Although it could not be proven that the combination treatment led to more frequent remissions, the study found significant and clinically relevant differences in multiple domains in favor of the intervention.

### 2.4 Therapies targeting the neonatal Fc receptor

The FcRn receptor is encoded by the FCGRT gene and is responsible for IgG homeostasis. In particular FcRn is responsible for the prevention of IgG degradation by recycling circulating IgG (67) (**Figure 1**). High-dose IVIG acts through multiple pathways, including competition with pathogenic autoantibodies for FcRn binding, which subsequently saturates the receptor and increases autoantibody clearance (43, 68, 69). It has been reported that patients with low FcRn expression may have a weaker response to IVIG treatment due to increased IVIG degradation (70).

Monoclonal antibodies against FcRn have been suggested to be effective at reducing serum pathogenic IgG autoantibody levels, without removing other circulating factors, such as albumin or clotting factors, or by affecting the complement pathway (3). Efgartigimod, a human IgG1 antibody Fc fragment, blocking the FcRn, has been shown to outcompete endogenous IgG binding, preventing IgG recycling. This subsequently reduces IgG and pathogenic IG autoantibody levels (71, 72). Efgartigimod has recently been approved by the Food and Drug Administration and the Japan’s Ministry of Health, Labour and Welfare as a treatment for CIDP based on positive results from the ADHERE trial, the largest clinical study to date on CIDP (**Figure 2**) (33, 34, 73). This phase 2, two-part, randomized, placebo-controlled trial (**Supplementary Table 1**) enrolled 330 adult patients with CIDP, assessing the efficacy, safety and tolerability of efgartigimod as a promising new approach to treating CIDP, to potentially help overcome the lack of innovative treatments for CIDP over the last three decades. Following a 12-week open-label phase, responders entered a 48-week randomized phase of weekly efgartigimod treatment vs. placebo. Primary objectives were evidenced by clinical response to treatment, and patients treated with efgartigimod remained relapse-free longer than those treated with placebo at stage B end of study (73.1% vs 46.4%, respectively) (34). Overall, efgartigimod offered convenience and potential advantages over traditional IVIG, including positive tolerability among patients, while simultaneously highlighting the significant role of IgG in CIDP and further insight into the disease pathogenesis (72, 74). In a real-world setting involving nine patients treated with efgartigimod, four experienced severe CIDP relapse and five showed no change (75). These data suggest that only those with predominant IgG autoantibody involvement may respond to IgG-lowering treatments like efgartigimod. Identifying CIDP biomarkers/subsets will be crucial to determine which patients will benefit the most from molecularly targeted treatments in a heterogeneous disease like CIDP.

Other FcRn inhibitors explored in CIDP include rozanolixizumab, a high-affinity human anti-FcRn IgG4 monoclonal antibody. In a phase 2 clinical trial (**Supplementary Table 1**) rozanolixizumab did not show efficacy in patients with CIDP, although this could be due to a relatively high placebo stability rate and absence of external diagnostic confirmation of CIDP (76). Nipocalimab, a fully human anti-FcRn glycosylated IgG1 monoclonal antibody; designed to selectively bind, saturate, and block the IgG binding site on the endogenous neonatal Fc receptor is also currently under investigation in a large multicenter clinical trial known as ARISE. This has a comparable trial design to the ADHERE trial, and plans to enroll 300 participants with an expected completion date in 2027 (77). Furthermore, batoclimab, another fully human anti-FcRn monoclonal antibody, has shown a potential role in the treatment of CIDP and is currently under investigation in randomized controlled trials (23, 78). Common adverse effects of FcRn therapy are similar to those of IG therapy, including injection-site reactions, infections, and headaches (34).

While mixed results are currently available for FcRn inhibitors, the high response rate in the ADHERE study suggests that autoantibodies are acting as the primary pathophysiological mechanism in a substantial proportion of patients with CIDP. Results from ongoing studies may help define the population for whom autoantibodies are driving CIDP.

### 2.5 Complement pathway inhibitors

Since therapies targeting humoral factors (plasma exchange and IVIG) are effective in patients with CIDP, autoantibodies and complement activation are considered key humoral effector mechanisms leading to demyelination in these patients (10). Autoantibodies may target the myelin, Schwann cell membranes or node of Ranvier structures leading to demyelination and axonal damage (79). Passive transfer of patient-derived serum or IgG can cause conduction block and demyelination in animal models, and an increase in complement activation (C3d) in the serum of patients suggests CIDP could also be complement-mediated (54, 79, 80).

Complement activation acts as a link between the adaptive and innate immune systems, through direct binding of receptors expressed at T cells, B cells, macrophages, and through modulation of dendritic cell function. The complement system has three different activation pathways, classical (C1q), lectin (mannose-binding lectins or ficolin), and the alternative (C3 autoactivation or properdin), which all converge at C3 (**Figure 1**). This generates the production of the effector proteins, C3a, C3b, C5a, and the membrane attack complex (MAC), C5b-9, which target cell lysis (10, 81). Preliminary studies have shown that targeting the complement system provides a promising new therapeutic strategy for CIDP (79).

The human monoclonal antibody riliprubart (a novel therapeutic agent which targets the classical complement pathway) has been shown to target active C1s protein, a C1 complex serine protease, which plays a key role in complement activation, and can selectively inhibit the C1-complex that prevents the activation of downstream enzymatic cascade that leads to C3 convertase activation and formation of MAC (**Supplementary Table 1**). This selective inhibition is responsible for blocking specific inflammatory mechanisms that lead to demyelination and axonal damage in CIDP (79). Positive preliminary results of a phase 2 trial determining the efficacy and safety of riliprubart in the treatment of CIDP were presented at the American Academy of Neurology annual meeting and at the PNS annual meeting in 2024, showing that 88% of patients improved or remained stable (interestingly, 52% of patients improved beyond their baseline status) after switching from SoC to riliprubart (35). Three participants relapsed (12%, n=3/25) while 50% SoC-refractory participants and 75% of treatment-naïve patients improved with riliprubart (35). Frequent adverse events reported in patients include headache, fatigue, and nasopharyngitis (35).

While the role of aberrant complement activation in CIDP pathology has been known for some time (54, 80), therapies that specifically target the complement mechanism of disease in CIDP are still in early development. Non-randomized evidence describes up to 88% of patients improving or remaining stable with riliprubart, suggesting complement may be a leading mode of disease within CIDP. The full results of the ongoing phase 2 trial for riliprubart may shed further light on the role of complement in CIDP.

### 2.6 Immunosuppressive treatments

Few clinical studies have been performed to evaluate the efficacy of other immunosuppressant agents for CIDP. Randomized studies of azathioprine, interferon beta-1a, fingolimod and methotrexate have reported no significant treatment response (82). Cyclophosphamide was used in a case series by Good et al., in a cohort of 15 participants who were all refractory to three initial first-line treatments. Complications of the treatment included nausea, vomiting and anemia. In combination with corticosteroids, this amelioration was achieved in four months. Cyclophosphamide is restricted in real-world settings for severe, refractory CIDP (83), and generally reserved for patients who fail to respond to conventional immunotherapy, and often taken in combination with prednisone (84). Although other immunomodulatory agents such as beta interferon have been reported to have beneficial effects, clinical trials have failed to confirm this benefit (22, 85, 86).

### 2.7 B-cell depletion therapy

Since the hypothesis that CIDP could be an autoantibody-mediated disorder, it has been postulated that therapies targeting B-cells responsible for the production of circulating pathogenic autoantibodies can be useful in CIDP. A prospective exploratory study using rituximab (an intravenous anti-CD20 monoclonal antibody) was conducted in 17 patients with CIDP who had not responded to at least two first-line therapies; overall, 76% of patients had an improvement of symptoms and no serious side effects were reported (36). Following this evidence, a randomized, double-blind, placebo-controlled trial that studied the effect of rituximab in delaying the need for IVIG reinfusion was carried out. Unfortunately, rituximab was not more effective in preventing clinical deterioration following the discontinuation of IG therapy in CIDP when compared with placebo (87, 88). Most adverse effects reported following rituximab therapy were mild and included limb pain, leukopenia, and a facial rash. However, some patients reported severe adverse events such as high fever and rash and clinical worsening due to IVIG delay (88). Despite the lack of efficacy rituximab has demonstrated in randomized controlled trials, observational studies continue to report positive outcomes in patients with CIDP. A retrospective cohort study identified that patients with CIDP who received combined, low-dose rituximab therapy presented with significantly reduced corticosteroid dosage and deterioration recurrence during follow-up, and a higher proportion of patients reported favorable response in scales assessments at each visit compared with patients who received conventional therapy (89). Similarly, a small study conducted in 15 patients with CIDP who received long-term low doses of rituximab found that 60% and 50% of patients exhibited significant clinical improvement compared to baseline evaluation following the first and second doses, respectively. Rituximab also had a favorable safety profile with no reported adverse events in this cohort (90). The use of rituximab to treat patients with CIDP remains a topic of debate. Although there is substantial positive data from non-randomized trials, indicating clinical improvement in most patients, randomized trials have not confirmed these findings. Given the current understanding of CIDP pathophysiology and the role of autoantibodies, B-cell depleting therapies may be considered as a treatment option. However, their use is likely to be limited to specific patient groups, which require precise definition.

## 3 Discussion

CIDP is a treatable yet disabling disorder, with a high response rate, but often suboptimal, to available first-line treatments including IVIG, SCIG, corticosteroids, plasma exchange and the recently approved FcRn inhibitor, efgartigimod. Since there are no established biomarkers for CIDP, clinical assessment remains the only evaluation tool for treatment selection and evaluation of efficacy. Therefore, the therapeutic management of CIDP varies for each patient, particularly those who are refractory or treatment naïve. The variable response to first-line treatments, and the notion that there are multiple phenotypic variations in clinical presentation, the treatment of patients with CIDP is also often complex, and no “one treatment suits all.” CIDP is a heterogeneous disease and thus requires a tailored therapeutic approach for individual patients. Results from single-targeting therapies may help identify biomarkers to guide optimum treatment decisions in the future.

Exploration of new therapeutic strategies has emerged in the last few years. In particular, the potential pathogenic roles of the complement pathway in CIDP have opened a new therapeutic window for drugs that inhibit complement activation. The FcRn receptor has also been shown as a potential pharmacological target, with the development of antibodies against FcRn that reduce circulating IgG and FcRn blockers, which competitively inhibit FcRn. Complement inhibition and FcRn saturation are among the mechanisms of action also seen in IVIG; therefore, it will be interesting to see how these single-target agents, once approved, will fit into the CIDP treatment landscape. Given the current proven options of IGs and corticosteroids, and the lack of robust biomarkers identifying subsets of patients with CIDP who are most likely to respond to specific agents are established, their uptake in clinical practice is hard to predict.

While more specific and individualized therapies are being developed, there is a need to increase the therapeutic landscape toward new drugs that target specific mechanism of disease pathways in CIDP, and biomarkers for monitoring treatment efficacy. Current data lean towards complement and autoantibodies being the primary drivers of pathophysiology in a substantial proportion of patients and therefore should be the focus of biomarker investigation.

## References

[1] Koike H Katsuno M. Pathophysiology of chronic inflammatory demyelinating polyneuropathy: insights into classification and therapeutic strategy. Neurol Ther. (2020) 9:213–27. doi: 10.1007/s40120-020-00190-8 PMC7606443 32410146

[2] Oaklander AL Lunn MP Hughes RA van Schaik IN Frost C Chalk CH. Treatments for chronic inflammatory demyelinating polyradiculoneuropathy (CIDP): an overview of systematic reviews. Cochrane Database Syst Rev. (2017) 1:CD010369. doi: 10.1002/14651858.CD010369.pub2 28084646 PMC5468847

[3] Briani C Visentin A. Therapeutic monoclonal antibody therapies in chronic autoimmune demyelinating neuropathies. Neurotherapeutics. (2022) 19:874–84. doi: 10.1007/s13311-022-01222-x PMC9294114 35349079

[4] Broers MC Bunschoten C Nieboer D Lingsma HF Jacobs BC. Incidence and prevalence of chronic inflammatory demyelinating polyradiculoneuropathy: A systematic review and meta-analysis. Neuroepidemiology. (2019) 52:161–72. doi: 10.1159/000494291 PMC6518865 30669140

[5] Rajabally YA Simpson BS Beri S Bankart J Gosalakkal JA. Epidemiologic variability of chronic inflammatory demyelinating polyneuropathy with different diagnostic criteria: study of a UK population. Muscle Nerve. (2009) 39:432–8. doi: 10.1002/mus.21206 19260065

[6] Gogia B Rocha Cabrero F Khan Suheb MZ Lui F Rai PK. Chronic Inflammatory Demyelinating Polyradiculoneuropathy. Treasure Island (FL: Statpearls (2024). 33085396

[7] Van den Bergh PYK van Doorn PA Hadden RDM Avau B Vankrunkelsven P Allen JA. European academy of neurology/peripheral nerve society guideline on diagnosis and treatment of chronic inflammatory demyelinating polyradiculoneuropathy: report of a joint task force-second revision. Eur J Neurol. (2021) 28:3556–83. doi: 10.1111/ene.14959 34327760

[8] Allen JA Lewis RA. CIDP diagnostic pitfalls and perception of treatment benefit. Neurology. (2015) 85:498–504. doi: 10.1212/WNL.0000000000001833 26180143

[9] Broers MC Bunschoten C Drenthen J Beck TAO Brusse E Lingsma HF. Misdiagnosis and diagnostic pitfalls of chronic inflammatory demyelinating polyradiculoneuropathy. Eur J Neurol. (2021) 28:2065–73. doi: 10.1111/ene.14796 PMC8252611 33657260

[10] Querol LA Hartung HP Lewis RA van Doorn PA Hammond TR Atassi N. The role of the complement system in chronic inflammatory demyelinating polyneuropathy: implications for complement-targeted therapies. Neurotherapeutics. (2022) 19:864–73. doi: 10.1007/s13311-022-01221-y PMC9294101 35378684

[11] Hagen KM Ousman SS. The immune response and aging in chronic inflammatory demyelinating polyradiculoneuropathy. J Neuroinflamm. (2021) 18:78. doi: 10.1186/s12974-021-02113-2 PMC7983397 33752693

[12] Chi LJ Xu WH Zhang ZW Huang HT Zhang LM Zhou J. Distribution of Th17 cells and Th1 cells in peripheral blood and cerebrospinal fluid in chronic inflammatory demyelinating polyradiculoneuropathy. J Peripher Nerv Syst. (2010) 15:345–56. doi: 10.1111/j.1529-8027.2010.00294.x 21199106

[13] Koike H Nishi R Ikeda S Kawagashira Y Iijima M Katsuno M. Ultrastructural mechanisms of macrophage-induced demyelination in CIDP. Neurology. (2018) 91:1051–60. doi: 10.1212/WNL.0000000000006625 30429275

[14] Mausberg AK Dorok M Stettner M Muller M Hartung HP Dehmel T. Recovery of the T-cell repertoire in CIDP by iv immunoglobulins. Neurology. (2013) 80:296–303. doi: 10.1212/WNL.0b013e31827debad 23269592

[15] Dalakas MC Engel WK. Immunoglobulin and complement deposits in nerves of patients with chronic relapsing polyneuropathy. Arch Neurol. (1980) 37:637–40. doi: 10.1001/archneur.1980.00500590061010 6252877

[16] Querol L Devaux J Rojas-Garcia R Illa I. Autoantibodies in chronic inflammatory neuropathies: diagnostic and therapeutic implications. Nat Rev Neurol. (2017) 13:533–47. doi: 10.1038/nrneurol.2017.84 28708133

[17] Pascual-Goni E Caballero-Avila M Querol L. Antibodies in autoimmune neuropathies: what to test, how to test, why to test. Neurology. (2024) 103:e209725. doi: 10.1212/WNL.0000000000209725 39088795 PMC11319070

[18] Janeway CA Jr Travers P Walport M Shlomchik MJ. Immunobiology: The Immune System in Health and Disease. 5th edition. New York: Garland Science (2001).

[19] Dalakas MC Medscape. Advances in the diagnosis, pathogenesis and treatment of CIDP. Nat Rev Neurol. (2011) 7:507–17. doi: 10.1038/nrneurol.2011.121 21844897

[20] Dalakas MC. Pathogenesis of immune-mediated neuropathies. Biochim Biophys Acta. (2015) 1852:658–66. doi: 10.1016/j.bbadis.2014.06.013 24949885

[21] Dalakas MC. Igg4-mediated neurologic autoimmunities: understanding the pathogenicity of Igg4, ineffectiveness of Ivig, and long-lasting benefits of anti-B cell therapies. Neurol Neuroimmunol Neuroinflamm. (2022) 9(1):e1116. doi: 10.1212/NXI.0000000000001116 34845096 PMC8630661

[22] Mahdi-Rogers M Rajabally YA. Overview of the pathogenesis and treatment of chronic inflammatory demyelinating polyneuropathy with intravenous immunoglobulins. Biologics. (2010) 4:45–9. doi: 10.2147/btt.s4881 PMC2846143 20376173

[23] Rajabally YA. Chronic inflammatory demyelinating polyradiculoneuropathy: current therapeutic approaches and future outlooks. Immunotargets Ther. (2024) 13:99–110. doi: 10.2147/ITT.S388151 38435981 PMC10906673

[24] Yoon MS Chan A Gold R. Standard and escalating treatment of chronic inflammatory demyelinating polyradiculoneuropathy. Ther Adv Neurol Disord. (2011) 4:193–200. doi: 10.1177/1756285611405564 21694819 PMC3105635

[25] Cocito D Paolasso I Antonini G Benedetti L Briani C Comi C. A nationwide retrospective analysis on the effect of immune therapies in patients with chronic inflammatory demyelinating polyradiculoneuropathy. Eur J Neurol. (2010) 17:289–94. doi: 10.1111/j.1468-1331.2009.02802.x 19863650

[26] Gorson KC van Schaik IN Merkies IS Lewis RA Barohn RJ Koski CL. Chronic inflammatory demyelinating polyneuropathy disease activity status: recommendations for clinical research standards and use in clinical practice. J Peripher Nerv Syst. (2010) 15:326–33. doi: 10.1111/j.1529-8027.2010.00284.x 21199104

[27] Al-Zuhairy A Jakobsen J. Outcome in chronic inflammatory demyelinating polyneuropathy: A systematic review and meta-analysis. Muscle Nerve. (2023) 68:388–96. doi: 10.1002/mus.27820 36928889

[28] Hughes RA Donofrio P Bril V Dalakas MC Deng C Hanna K. Intravenous immune globulin (10% Caprylate-chromatography purified) for the treatment of chronic inflammatory demyelinating polyradiculoneuropathy (Ice study): A randomised placebo-controlled trial. Lancet Neurol. (2008) 7:136–44. doi: 10.1016/S1474-4422(07)70329-0 18178525

[29] Bril V Hadden RDM Brannagan TH 3rd Bar M Chroni E Rejdak K. Hyaluronidase-facilitated subcutaneous immunoglobulin 10% as maintenance therapy for chronic inflammatory demyelinating polyradiculoneuropathy: the advance-Cidp 1 randomized controlled trial. J Peripher Nerv Syst. (2023) 28:436–49. doi: 10.1111/jns.12573 37314318

[30] van Schaik IN Bril V van Geloven N Hartung HP Lewis RA Sobue G. Subcutaneous immunoglobulin for maintenance treatment in chronic inflammatory demyelinating polyneuropathy (Path): A randomised, double-blind, placebo-controlled, phase 3 trial. Lancet Neurol. (2018) 17:35–46. doi: 10.1016/S1474-4422(17)30378-2 29122523

[31] Hadden RDM Andersen H Bril V Basta I Rejdak K Duff K. Long-term safety and tolerability of hyaluronidase-facilitated subcutaneous immunoglobulin 10% as maintenance therapy for chronic inflammatory demyelinating polyradiculoneuropathy: results from the advance-cidp 3 trial. J Peripher Nerv Syst. (2024) 29:441–52. doi: 10.1111/jns.12672 PMC11625974 39523874

[32] Austin JH. Recurrent polyneuropathies and their corticosteroid treatment; with five-year observations of a placebo-controlled case treated with corticotrophin, cortisone, and prednisone. Brain. (1958) 81:157–92. doi: 10.1093/brain/81.2.157 13572689

[33] argenx Announces Approval of VYVDURA (efgartigimod alfa and hyaluronidase-qvfc) in Japan for Adults with Chronic Inflammatory Demyelinating Polyneuropathy. (2024). Available online at: https://argenx.com/news/2024/argenx-announces-approval-of-vyvdura--efgartigimod-alfa-and-hyal.

[34] Allen JA Lin J Basta I Dysgaard T Eggers C Guptill JT. Safety, tolerability, and efficacy of subcutaneous efgartigimod in patients with chronic inflammatory demyelinating polyradiculoneuropathy (Adhere): A multicentre, randomised-withdrawal, double-blind, placebo-controlled, phase 2 trial. Lancet Neurol. (2024) 23:1013–24. doi: 10.1016/S1474-4422(24)00309-0 39304241

[35] Querol L Lewis R Hartung H Van Doorn P Wallstroem E Luo X. Preliminary efficacy and safety data from the phase 2 trial of riliprubart (Sar445088), a humanized monoclonal antibody targeting complement C1s, in chronic inflammatory demyelinating polyneuropathy (Cidp) (S15.008). Neurology. (2024) 102:supplement_1. doi: 10.1212/WNL.0000000000204596

[36] Doneddu PE Cocito D Fazio R Benedetti L Peci E Liberatore G. Prospective open-label trial with rituximab in patients with chronic inflammatory demyelinating polyradiculoneuropathy not responding to conventional immune therapies. J Neurol Neurosurg Psychiatry. (2024) 95:838–44. doi: 10.1136/jnnp-2023-332844 38729746

[37] Server AC Lefkowith J Braine H McKhann GM. Treatment of chronic relapsing inflammatory polyradiculoneuropathy by plasma exchange. Ann Neurol. (1979) 6:258–61. doi: 10.1002/ana.410060313 534424

[38] Briani C Zara G Zambello R Trentin L Rana M Zaja F. Rituximab-responsive CIDP. Eur J Neurol. (2004) 11:788. doi: 10.1111/j.1468-1331.2004.00911.x 15525302

[39] Markvardsen LH Debost JC Harbo T Sindrup SH Andersen H Christiansen I. Subcutaneous immunoglobulin in responders to intravenous therapy with chronic inflammatory demyelinating polyradiculoneuropathy. Eur J Neurol. (2013) 20:836–42. doi: 10.1111/ene.12080 23294032

[40] van Doorn PA Brand A Strengers PF Meulstee J Vermeulen M. High-dose intravenous immunoglobulin treatment in chronic inflammatory demyelinating polyneuropathy: A double-blind, placebo-controlled, crossover study. Neurology. (1990) 40:209–12. doi: 10.1212/wnl.40.2.209 2405291

[41] Dalakas MC. The use of intravenous immunoglobulin in the treatment of autoimmune neuromuscular diseases: evidence-based indications and safety profile. Pharmacol Ther. (2004) 102:177–93. doi: 10.1016/j.pharmthera.2004.04.002 15246245

[42] Dalakas MC Latov N Kuitwaard K. Intravenous immunoglobulin in chronic inflammatory demyelinating polyradiculoneuropathy (CIDP): mechanisms of action and clinical and genetic considerations. Expert Rev Neurother. (2022) 22:953–62. doi: 10.1080/14737175.2022.2169134 36645654

[43] Bleeker WK Teeling JL Hack CE. Accelerated autoantibody clearance by intravenous immunoglobulin therapy: studies in experimental models to determine the magnitude and time course of the effect. Blood. (2001) 98:3136–42. doi: 10.1182/blood.v98.10.3136 11698302

[44] Seite JF Goutsmedt C Youinou P Pers JO Hillion S. Intravenous immunoglobulin induces a functional silencing program similar to anergy in human B cells. J Allergy Clin Immunol. (2014) 133:181–8.e1-9. doi: 10.1016/j.jaci.2013.08.042 24139609

[45] Dolezal O. Intravenous immunoglobulin treatment in chronic neurological diseases: do we have maintenance dose right? Autoimmune Dis. (2014) 2014:962530. doi: 10.1155/2014/962530 25580286 PMC4281444

[46] Rajabally YA Afzal S. Clinical and economic comparison of an individualised immunoglobulin protocol vs. Standard dosing for chronic inflammatory demyelinating polyneuropathy. J Neurol. (2019) 266:461–7. doi: 10.1007/s00415-018-9157-4 PMC6373347 30556098

[47] Nobile-Orazio E Pujol S Kasiborski F Ouaja R Corte GD Bonek R. An international multicenter efficacy and safety study of iqymune in initial and maintenance treatment of patients with chronic inflammatory demyelinating polyradiculoneuropathy: prism study. J Peripher Nerv Syst. (2020) 25:356–65. doi: 10.1111/jns.12408 PMC7754365 32808406

[48] Mendell JR Barohn RJ Freimer ML Kissel JT King W Nagaraja HN. Randomized controlled trial of Ivig in untreated chronic inflammatory demyelinating polyradiculoneuropathy. Neurology. (2001) 56:445–9. doi: 10.1212/wnl.56.4.445 11222785

[49] Vermeulen M van Doorn PA Brand A Strengers PF Jennekens FG Busch HF. Intravenous immunoglobulin treatment in patients with chronic inflammatory demyelinating polyneuropathy: A double blind, placebo controlled study. J Neurol Neurosurg Psychiatry. (1993) 56:36–9. doi: 10.1136/jnnp.56.1.36 PMC1014761 8429321

[50] Thompson N Choudhary P Hughes RA Quinlivan RM. A novel trial design to study the effect of intravenous immunoglobulin in chronic inflammatory demyelinating polyradiculoneuropathy. J Neurol. (1996) 243:280–5. doi: 10.1007/BF00868527 8936360

[51] Hahn AF Bolton CF Zochodne D Feasby TE. Intravenous immunoglobulin treatment in chronic inflammatory demyelinating polyneuropathy. A double-blind, placebo-controlled, cross-over study. Brain. (1996) 119:1067–77. doi: 10.1093/brain/119.4.1067 8813271

[52] Kuwabara S Mori M Misawa S Suzuki M Nishiyama K Mutoh T. Intravenous immunoglobulin for maintenance treatment of chronic inflammatory demyelinating polyneuropathy: A multicentre, open-label, 52-week phase iii trial. J Neurol Neurosurg Psychiatry. (2017) 88:832–8. doi: 10.1136/jnnp-2017-316427 PMC5629934 28768822

[53] Mehndiratta MM Hughes RA Pritchard J. Plasma exchange for chronic inflammatory demyelinating polyradiculoneuropathy. Cochrane Database Syst Rev. (2015) 2015:CD003906. doi: 10.1002/14651858.CD003906.pub4 26305459 PMC6734114

[54] Heininger K Liebert UG Toyka KV Haneveld FT Schwendemann G Kolb-Bachofen V. Chronic inflammatory polyneuropathy. Reduction of nerve conduction velocities in monkeys by systemic passive transfer of immunoglobulin G. J Neurol Sci. (1984) 66:1–14. doi: 10.1016/0022-510x(84)90136-9 6394721

[55] Hahn AF Bolton CF Pillay N Chalk C Benstead T Bril V. Plasma-exchange therapy in chronic inflammatory demyelinating polyneuropathy. A double-blind, sham-controlled, cross-over study. Brain. (1996) 119:1055–66. doi: 10.1093/brain/119.4.1055 8813270

[56] Dyck PJ Daube J O’Brien P Pineda A Low PA Windebank AJ. Plasma exchange in chronic inflammatory demyelinating polyradiculoneuropathy. N Engl J Med. (1986) 314:461–5. doi: 10.1056/NEJM198602203140801 3511382

[57] Ripellino P Fleetwood T Cantello R Comi C. Treatment of chronic inflammatory demyelinating polyneuropathy: from molecular bases to practical considerations. Autoimmune Dis. (2014) 2014:201657. doi: 10.1155/2014/201657 24527207 PMC3914592

[58] Losel RM Falkenstein E Feuring M Schultz A Tillmann HC Rossol-Haseroth K. Nongenomic steroid action: controversies, questions, and answers. Physiol Rev. (2003) 83:965–1016. doi: 10.1152/physrev.00003.2003 12843413

[59] Hughes RA Mehndiratta MM Rajabally YA. Corticosteroids for chronic inflammatory demyelinating polyradiculoneuropathy. Cochrane Database Syst Rev. (2017) 11:CD002062. doi: 10.1002/14651858.CD002062.pub4 29185258 PMC6747552

[60] Flammer JR Rogatsky I. Minireview: glucocorticoids in autoimmunity: unexpected targets and mechanisms. Mol Endocrinol. (2011) 25:1075–86. doi: 10.1210/me.2011-0068 PMC5417249 21511881

[61] Rabin M Mutlu G Stojkovic T Maisonobe T Lenglet T Fournier E. Chronic inflammatory demyelinating polyradiculoneuropathy: search for factors associated with treatment dependence or successful withdrawal. J Neurol Neurosurg Psychiatry. (2014) 85:901–6. doi: 10.1136/jnnp-2013-306105 24309269

[62] Nobile-Orazio E Cocito D Jann S Uncini A Messina P Antonini G. Frequency and time to relapse after discontinuing 6-month therapy with Ivig or pulsed methylprednisolone in CIDP. J Neurol Neurosurg Psychiatry. (2015) 86:729–34. doi: 10.1136/jnnp-2013-307515 25246645

[63] van Schaik IN Eftimov F van Doorn PA Brusse E van den Berg LH van der Pol WL. Pulsed high-dose dexamethasone versus standard prednisolone treatment for chronic inflammatory demyelinating polyradiculoneuropathy (Predict study): A double-blind, randomised, controlled trial. Lancet Neurol. (2010) 9:245–53. doi: 10.1016/S1474-4422(10)70021-1 20133204

[64] van Lieverloo GGA Peric S Doneddu PE Gallia F Nikolic A Wieske L. Corticosteroids in chronic inflammatory demyelinating polyneuropathy: A retrospective, multicentre study, comparing efficacy and safety of daily prednisolone, pulsed dexamethasone, and pulsed intravenous methylprednisolone. J Neurol. (2018) 265:2052–9. doi: 10.1007/s00415-018-8948-y PMC6132640 29968199

[65] Bus SRM Zambreanu L Abbas A Rajabally YA Hadden RDM de Haan RJ. Intravenous immunoglobulin and intravenous methylprednisolone as optimal induction treatment in chronic inflammatory demyelinating polyradiculoneuropathy: protocol of an international, randomised, double-blind, placebo-controlled trial (Optic). Trials. (2021) 22:155. doi: 10.1186/s13063-021-05083-1 33608058 PMC7894234

[66] van Doorn I Bus S Zambreanu L Abbas A Rajabally Y Hadden R. Optic trial: intravenous immunoglobulin and intravenous methylprednisolone as induction treatment in CIDP. PNS Annual Meeting (2024) S190. doi: 10.1111/jns.12648

[67] Roopenian DC Akilesh S. Fcrn: the neonatal fc receptor comes of age. Nat Rev Immunol. (2007) 7:715–25. doi: 10.1038/nri2155 17703228

[68] Masson PL. Elimination of infectious antigens and increase of igg catabolism as possible modes of action of Ivig. J Autoimmun. (1993) 6:683–9. doi: 10.1006/jaut.1993.1057 8155250

[69] Yu Z Lennon VA. Mechanism of intravenous immune globulin therapy in antibody-mediated autoimmune diseases. N Engl J Med. (1999) 340:227–8. doi: 10.1056/NEJM199901213400311 9895405

[70] Fisse AL Schafer E Hieke A Schroder M Klimas R Brunger J. Association of the neonatal fc receptor promoter variable number of tandem repeat polymorphism with immunoglobulin response in patients with chronic inflammatory demyelinating polyneuropathy. Eur J Neurol. (2024) 31:e16205. doi: 10.1111/ene.16205 38205888 PMC11235998

[71] Lewis R. Results on efgartigimod for chronic inflammatory demyelinating polyneuropathy treatment: Richard Lewis, Md(2023). Available online at: https://www.neurologylive.com/view/results-efgartigimod-cidp-treatment-richard-lewis (Accessed November 3, 2023).

[72] Allen J Basta I Eggers C Guptill J Gwathmey K Hewamadduma C. Efficacy, Safety, and Tolerability of Efgartigimod in Patients with Chronic Inflammatory Demyelinating Polyneuropathy: results from the ADHERE Trial. Cochrane Library. Neurology. (2024) 102(17). doi: 10.1212/WNL.0000000000206324

[73] Argenx Announces Approval of Vyvdura (Efgartigimod Alfa and Hyaluronidase-Qvfc) in Japan for Adults with Chronic Inflammatory Demyelinating Polyneuropathy (2024). Available otline at: https://argenx.com/news/2024/argenx-announces-approval-of-vyvdura--efgartigimod-alfa-and-hyal.

[74] Allen J De Haard H Parys W Ulrichts P Guglietta A Hofman E. Efgartigimod in Chronic Inflammatory Demyelinating Polyneuropathy: Adhere Phase 2 Trial Design. Cochrane Library (2021). doi: 10.1002/central/CN-02261268/full.

[75] Levine T Muley S. Early deterioration of CIDP following transition from Ivig to Fcrn inhibitor treatment. J Neurol Sci. (2024) 468:123313. doi: 10.1016/j.jns.2024.123313 39578164

[76] Querol L De Seze J Dysgaard T Levine T Rao TH Rivner M. Efficacy, safety and tolerability of rozanolixizumab in patients with chronic inflammatory demyelinating polyradiculoneuropathy: A randomised, subject-blind, investigator-blind, placebo-controlled, phase 2a trial and open-label extension study. J Neurol Neurosurg Psychiatry. (2024) 95:845–54. doi: 10.1136/jnnp-2023-333112 PMC11347201 38729747

[77] ClinicalTrials.gov. Efficacy and safety study of nipocalimab for adults with chronic inflammatory demyelinating polyneuropathy (Cidp) (2022). Available online at: https://classic.clinicaltrials.gov/ct2/show/NCT05327114 (Accessed March 25, 2025).

[78] ClinicalTrials.gov. To assess efficacy and safety of batoclimab in adult participants with active Cidp (2022). Available online at: https://classic.clinicaltrials.gov/ct2/show/NCT05581199 (Accessed November 25, 2025).

[79] Querol L Lewis RA Hartung HP Van Doorn PA Wallstroem E Luo X. An innovative phase 2 proof-of-concept trial design to evaluate sar445088, a monoclonal antibody targeting complement C1s in chronic inflammatory demyelinating polyneuropathy. J Peripher Nerv Syst. (2023) 28:276–85. doi: 10.1111/jns.12551 37119056

[80] Yan WX Archelos JJ Hartung HP Pollard JD. P0 protein is a target antigen in chronic inflammatory demyelinating polyradiculoneuropathy. Ann Neurol. (2001) 50:286–92. doi: 10.1002/ana.1129 11558784

[81] Dalakas MC Alexopoulos H Spaeth PJ. Complement in neurological disorders and emerging complement-targeted therapeutics. Nat Rev Neurol. (2020) 16:601–17. doi: 10.1038/s41582-020-0400-0 PMC7528717 33005040

[82] Fisse AL Motte J Gruter T Sgodzai M Pitarokoili K Gold R. Comprehensive approaches for diagnosis, monitoring and treatment of chronic inflammatory demyelinating polyneuropathy. Neurol Res Pract. (2020) 2:42. doi: 10.1186/s42466-020-00088-8 33324942 PMC7722337

[83] Good JL Chehrenama M Mayer RF Koski CL. Pulse cyclophosphamide therapy in chronic inflammatory demyelinating polyneuropathy. Neurology. (1998) 51:1735–8. doi: 10.1212/wnl.51.6.1735 9855536

[84] Gorson KC. An update on the management of chronic inflammatory demyelinating polyneuropathy. Ther Adv Neurol Disord. (2012) 5:359–73. doi: 10.1177/1756285612457215 PMC3487533 23139706

[85] Choudhary PP Thompson N Hughes RA. Improvement following interferon beta in chronic inflammatory demyelinating polyradiculoneuropathy. J Neurol. (1995) 242:252–3. doi: 10.1007/BF00919601 7798127

[86] Hadden RD Sharrack B Bensa S Soudain SE Hughes RA. Randomized trial of interferon beta-1a in chronic inflammatory demyelinating polyradiculoneuropathy. Neurology. (1999) 53:57–61. doi: 10.1212/wnl.53.1.57 10408537

[87] Nobile-Orazio E Cocito D Manganelli F Fazio R Lauria G. A randomiezed controlled trial with rituixmab to prevent linical worsening in CIDP after immunoglobulin suspension. PNS annual meeting 2024 abstract supplement. Abstact supplement (2024) S186. doi: 10.1111/jns.12648

[88] Nobile-Orazio E Cocito D Manganelli F Fazio R Lauria Pinter G Benedetti L. Rituximab versus placebo for chronic inflammatory demyelinating polyradiculoneuropathy: A randomized trial. Brain. (2024) 10:awae400. doi: 10.1093/brain/awae400 PMC11967823 39658326

[89] Du Y Yan Q Li C Zhu W Zhao C Hao Y. Efficacy and safety of combined low-dose rituximab regimen for chronic inflammatory demyelinating polyradiculoneuropathy. Ann Clin Transl Neurol. (2025) 12(1):180–91. doi: 10.1002/acn3.52270 PMC11752089 39660535

[90] Zheng Y Sun C Zhao Y Meng Q Hu J Qiao K. Long-term and low-dose rituximab treatment for chronic inflammatory demyelinating polyneuropathy. J Peripher Nerv Syst. (2024) 29:350–5. doi: 10.1111/jns.12653 39152723

