# Supplementary material for: A pathophysiological and mechanistic review of chronic inflammatory demyelinating polyradiculoneuropathy therapy
Source: Front Immunol. 2025 Apr 14;16:1575464. doi: 10.3389/fimmu.2025.1575464 (PMC12034639; doi:10.3389/fimmu.2025.1575464)
Supplement: Supplementary file 1 [file Table1.docx]

**Supplementary Table 1. Summary of key studies evaluating CIDP treatments (1-26)**.

| **Study name** | **NCT number** | **Interventions** | **Study Design** | | **Patient (n)** | | **Endpoints** | | **Key results** | **Duration** | **Reference** |
| --- | --- | --- | --- | --- | --- | --- | --- | --- | --- | --- | --- |
| **Immunoglobulin treatment** | | | | | | | | | | | |
| ***IVIG*** | | | | | | | | | | | |
| **Intravenous Immunoglobulin Treatment in Patients with Chronic Inflammatory Demyelinating Polyneuropathy** | N/A | High-dose IVIG of 0.4 g/kg of body weight per day for five consecutive days | Prospective, controlled trial | | 52 | | **Primary endpoints:**   - Muscle strength using the MRC scale for six muscle groups - Disability was assessed using the Rankin scale; improvement was defined as an increase of at least one step on the scale | | **Primary outcomes:**   - 62% (n=32/52) patients improved after IVIG and was maintained in 90% of patients for a median follow-up period of 4 years - Four patients improved one grade, 10 improved two grades, eight improved three grades, and 10 patients improved four grades on the Rankin scale | 5 days | van Doorn P.A., *et al. Arch Neurol.* 1991;48:217–220 |
| **IVIG in CIDP: a double-blind, placebo-controlled study** | N/A | IVIG vs placebo  Freeze-dried IVIG  0.4 g/kg bodyweight/day for 5 consecutive days | Randomized, double-blind, placebo-controlled, multicenter trial | | 28 | | **Primary endpoints:**   - Degree of improvement between IVIG and placebo group using a six-point Rankin scale   **Secondary endpoints:**   - Muscle weakness of arms and legs using the MRC scale | | **Primary outcomes:**   - 27% of patients in IVIG improved by at least one point on the Rankin scale compared with 23% in placebo   **Secondary outcomes:**  Degree of improvement of MRC sum score was similar between groups | 3 weeks | Vermeulen M, *et al. Journal of Neurol, Neuro, and Psych.* 1993;56:36–39 |
| **Intravenous immunoglobulin treatment in chronic inflammatory demyelinating polyneuropathy A double-blind, placebo-controlled, cross-over study** | N/A | IVIG vs placebo IVIG 0.4 g per kg body weight or a placebo treatment | Double-blind, placebo-controlled, cross-over study | | 30 | | **Primary endpoints:**   - Neurological function, measured by NDS, CG, GS, and electrophysiological studies before and after each treatment period - A secondary two-group analysis of the first trial period was also performed | | **Primary outcomes:**   - Significant differences in favor of IVIG were observed; patients had improved NDS (24.4±5.4 points; P<0.002), CG (1.0±0.3 points; P<0.001), and GS (+6.3±1.7 kg; P<0.005) - Scores were unchanged or worse in patients who received placebo   **Secondary two-group analysis**   - Significant differences in favor of IVIG were observed; patients had improved NDS (35.6±25.0 points; P<0.0001), CG (1.3±1.9 points; P<0.002), and GS (+9.8±7.7 kg; P<0.001) - All scores worsened in placebo - 63% of patients with CIDP improved on IVIG - Statistically significant improvements in the summed motor conduction velocities | 5 days | Hahn A.F., *et al*. *Brain.* 1996;119:1067–1077 |
| **A novel trial design to study the effect of intravenous immunoglobulin in chronic inflammatory demyelinating polyradiculoneuropathy** | N/A | IVIG vs placebo  Arm 1: IVIG 0.4 g/kg body weight per day or albumin solution of similar appearance for five consecutive days  Arm 2: IVIG 0.4 g/kg daily for five days (open fashion) and then randomized to either receive IVIG 0.4 g/kg every 3 weeks or albumin every 3 weeks | Double-blind, placebo-controlled, cross-over study | | 7 | | **Primary endpoints:**   - Neuromuscular function assessed using an expanded version of the MRC sum score and a shortened version of the Hammersmith Motor Ability Score, nine-hole peg test, a timed 10 m walk and the Ambulation Index - EMG examination | | **Primary outcomes:**   - Three patients showed a significant response to IVIG infusion; the remaining four showed no clear differential response - EMG parameters showed significant change in any patient - Only one patient entered the second arm of the trial and was soon deemed to need open IVIG due to deterioration of their functional ability | Arm 1: 4 weeks  Arm 2: 24 weeks | Thompson N, *et al. J Neurol*. 1996;243:280–285 |
| **Randomized controlled trial of IVIG in untreated CIDP** | N/A | IVIG vs placebo  IVIG 5% albumin 1 g/kg | Randomized, controlled trial | | 33 | | **Primary endpoints:**   - Change in muscle strength from baseline to day 43 using AMS   **Secondary endpoints:**   - Change from baseline AMS at days 10 and 21 - Hughe’s functional disability scale - FVC - NCS of the median, ulnar, peroneal, and tibial motor nerves | | **Primary outcomes:**   - Baseline AMS values were similar between groups - Mean AMS significantly improved compared with placebo (p=0.006)   **Secondary outcomes:**   - IVIG group had significantly improved by the functional disability scale compared with placebo group (p=0.019) - FVC did not improve in either group - IVIG improved NCS of motor nerves | 6 weeks | Mendell J.R., *et al*. *Neurol.* 2001 Feb 27;56(4):445-449 |
| **ICE** | 00220740 | IVIG vs placebo  IVIG 10% caprylate-chromatography purified (Gamunex)  1 g/kg over  1–2 days every 3 weeks  1:1 | Phase III, randomized, double blinded, placebo controlled, response-conditional crossover trial | | 117 | | **Primary endpoints:**   - Responder rate between IVIG-C (Gamunex) and placebo, as defined by INCAT disability score | | **Primary outcomes:**   - 54% of patients treated with IVIG-C had an improved in adjusted INCAT disability score that was maintained for 24 weeks | 24 weeks | Hughes R.A., *et al*. *Lancet Neurol*. 2008;7(2):136–144 |
| **PRIMA** | 01184846 | IVIG (Privigen) 2 g/kg (induction dose) then 1 g/kg every 3 weeks | Phase III, open label single arm trial | | 28 | | **Primary endpoints:**   - Responder rate between IVIG (Privigen) and placebo at EOS, as defined by INCAT disability score   **Secondary endpoints:**   - Grip strength - MRC sum score - AEs | | **Primary outcomes:**   - Response rate IVIG: 61% - IVIG pre-treated patients: 77% - naïve: 47% - INCAT score improved from 3.5 points at baseline to 2.5 points at EOS - Secondary outcomes: - Grip strength: 66.7 to 80.9 kPa - MRC score: 67.0 to 75.5 - AE: 88% were mild/moderate | 25 weeks | Léger J.M., *et al*. *J* *Peripher Nerv Syst*. 2013;18(2):130–140 |
| **Intravenous immunoglobulin for maintenance treatment of chronic inflammatory demyelinating polyneuropathy: a multicentre, open-label, 52-week phase III trial** | NCT01824251 | After an induction IVIG (0.4 g/kg/day for five consecutive days), maintenance IVIG (1.0 g/kg) was given every 3 weeks for up to 52 weeks. | Phase III, open-label trial conducted in 49 Japanese tertiary centers | | 49 | | **Primary endpoints:**   - Responder rate at week 28 - Relapse rate at week 52   **Secondary endpoints:**   - INCAT score - ISS score - Hand-grip strength - Serum IgG level - Number of days taken to improve the INCAT scare - Number of days until recurrence - Safety | | **Primary outcomes:**   - At week 28, 77.6% of patients experienced sustained INCAT score improvement of ≥1 points compared with their score at week one - From week 29 to 52, 10.5% of patients relapsed   **Secondary outcomes:**   - The mean value of all parameters improved from baseline to week 28 and was maintained upto week 52 - Serum IgG levels were higher at week four than baseline and were maintained at ~2000 mg/dL - Median number of days taken for the INCAT score to improve by 1 point was 45.0 days; improvement rate was 97% - 94% experienced adverse events, including headaches (33%), nasopharyngitis (29%), and skin rash (12%) | 52 weeks | Kuwabara S. *et al. J Neurol Neurosurg Psychiatry.* 2017;88:832–838 |
| **PRISM** | 02293460 | IVIG (IqYmune)  1 g/kg every  3 weeks | Phase III, multicenter, open label prospective single arm trial | | 44 | | **Primary endpoints:**   - Responder rate between IVIG (IqYmune) and historical placebo as defined as INCAT disability score   **Secondary endpoints:**   - Time to response at EOS - Grip strength - MRC sum score - AEs | | **Primary outcomes:**   - Overall response rate IVIG: 76%. Experienced: 84%. Naïve: 70%   **Secondary outcomes:**   - Median time to response of responders was 15 weeks (response occurred earlier in Ig-pre-treated patients with 7.9 weeks) - All other scores were statistical improvement from baseline - AEs: 87.2% classified as mild | 24 weeks | Nobile-Orazio E, *et al*. J *Peripher Nerv Syst*. 2020 Dec;25(4):356–365 |
| **ProCID** | 02638207 | Randomized 1:2:1 of 0.5 or 1 or 2 g/kg IVIG (NewGam) every 3 weeks | Phase III, prospective double blinded, parallel trial | | 139 | | **Primary endpoints:**   - Responder rate between IVIG (NewGam) at 0.5/1.0/2.0 g/kg vs. placebo as defined by INCAT disability score   **Secondary endpoints:**   - Dose response and safety | | **Primary outcomes:**   - Response rate at:   0.5 g/kg: 65%  1 g/kg: 80%  2 g/kg: 92%  **Secondary outcomes:**   - AEs were reported in patient groups:   0.5 g/kg: 45.7%  1 g/kg: 46.4%  2 g/kg: 52.6% | 24 weeks | Cornblath D.R., *et al*. *Brain*. 2022;145(3):887–896 |
| ***SCIG*** | | | | | | | | | | | |
| **Subcutaneous immunoglobulin in responders to intravenous therapy with chronic inflammatory demyelinating polyradiculoneuropathy** | 01017159 | SCIG vs placebo (subcutaneous saline)  SCIG was administered at a dose corresponding to the patient’s pre-study IVIG dose or subcutaneous saline was given twice or thrice weekly for 12 weeks at home | Randomized, double-blind, placebo-controlled trial | | 30 | | **Primary endpoints:**   - Isokinetic strength performance of four predetermined and weakened muscle groups   **Secondary endpoints:**   - ODSS - 40-MWT - Nine-hole-peg test - NIS score - MRC score - Grip strength - Standardized electrophysiological recordings from three nerves - Plasma IgG levels | | **Primary outcomes:**   - In the SCIG group there was an increase of isokinetic muscle strength of 5.5±9.5% (P<0.05) compared with a decline of 14.4±20.3% (p<0.05) in the placebo group   **Secondary outcomes:**   - ODSS, NIS, MRC, grip strength and 40-MWT improved following SCIG vs placebo | 12 weeks | Markvardsen L.H., *et al*. *Eur J Neurol.* 2013;20(5): 836–842 |
| **Subcutaneous immunoglobulin as first-line therapy in treatment-naïve patients with chronic inflammatory demyelinating polyneuropathy:randomized controlled trial study** | [EudraCT] 2013-001428-20) | SCIG vs IVIG  0.4 g/kg/week SCIG for five weeks or 0.4 g/kg/day IVIG for five days  After 10 weeks, patients were switched to the opposite treatment arm | Randomized, double-blind, cross-over trial | | 20 | | **Primary endpoints:**   - Isokinetic muscle strength   **Secondary endpoints:**   - Disability - Clinical evaluation of muscle strength - Performance of various function tests | | **Primary outcomes:**   - Isokinetic muscle strength increased by 7.4±14.5% (p=0.0003) during SCIG and by 6.9±16.8% (p=0.002) during IVIG - Isokinetic strength peaked at two weeks after IVIG and five weeks after SCIG   **Secondary outcomes:**   - Disability improved during SCIG only - Muscle strength improved after five and 10 weeks during SCIG but only after five weeks with IVIG - Remaining parameters improved equally during both treatments | 20 weeks | Markvardsen L.H., *et al. Eur J Neurol.*2017;0:412–418 |
| **PATH** | [01545076](http://clinicaltrials.gov/show/NCT01545076) | SCIG (IgPro20) vs. placebo  0.2 g/kg or 0.4 g/kg of 20% SCIG solution (IgPro20) weekly or placebo  1:1:1 | Phase III, randomized double-blinded, placebo-controlled trial | | 172 | | **Primary endpoints:**   - Proportion of patients with a CIDP relapse or were withdrawn from the study | | **Primary outcomes:**   - Placebo patients: 63% - Patients on low dose SCIG: 39% - Patients on high dose SCIG: 33% had a relapse or were withdrawn from the study | 24 weeks | van Schaik I.N., *et al*. *Lancet Neurol*. 2018;17(1): 35–46 |
| **ADVANCE** | 02549170 | fSCIG (with recombinant human hyaluronidase) vs placebo to receive either fSCIG 10% or placebo  1:1 | Phase III, randomized double blinded, multicenter, placebo-controlled trial | | 132 | | **Primary endpoints:**   - CIDP relapse as defined by adjusted INCAT score   **Secondary outcome:**   - Safety endpoints | | **Primary outcomes:**   - Relapse fSCIG: 10% - Relapse placebo: 31% - Relapse probability was higher with placebo vs. fSCIG 10% over time   **Secondary outcomes:**   - AEs were more frequent with fSCIG 10% than placebo | 24 weeks | Bril V, *et al*. *J Peripher Nerv Syst*. 2023;28(3): 436–449  Hadden R.D.M., *et al. J Peripher Nerv Syst.* 2024;29(4): 441–452 |
| **Plasma Exchange** | | | | | | | | | | | |
| **Plasma exchange in chronic inflammatory demyelinating polyradiculoneuropathy** | N/A | Plasma or sham exchange administered twice a week for 3 weeks | Prospective, double-blind trial | 29 | | **Primary endpoints:**   - Neurologic-disability score, including summed score of muscle strength, reflexes, and sensory loss - Dynamometer measurement of maximal hand grip - Maximal inspiratory and expiratory pressure - Determination of the thresholds at which touch pressure could be detected by the big toe and thermal cooling by the dorsal foot | | **Primary outcomes:**   - Statistically significant differences were observed in combined measurements of nerve conduction, favoring patients who received plasma exchange. Five patients receiving plasma exchange had significantly greater improvement in disability scores (P=0.025) compared with sham exchange patients | | 3 weeks | Dyck P.J., *et al. NEJM*. 1986;314(8):461–465. |
| **Double-blind, sham-controlled, cross-over study** | N/A | 10 plasma-exchange or sham plasma exchange over 4 weeks | Double-blind, sham-controlled trial | 18 | | **Primary endpoints:**   - Neurological disability score - Functional clinical grade - Grip strength | | **Primary outcomes:**   - Patients receiving plasma exchange showed significant improvement in all clinical outcomes compared with sham plasma-exchange | | 9 weeks | Hahn A.F., *et al. Brain.* 1996 August;119(4):1055–1066 |
| **Corticosteroids** | | | | | | | | | | | |
| **Prednisone improves chronic inflammatory demyelinating polyradiculoneuropathy more than no treatment** | N/A | Prednisone vs no treatment  Prednisone began at a dosage of 120 mg every second day. On alternate days, except for weeks 11 and 12 when nothing was given, patients were given 5 mg | Randomized, controlled trial | | 40 | | **Primary endpoints:**   - NDS score before onset of study, at six weeks, and at three months after treatment - Maximum expiratory and inspiratory breathing measures - Strength of handgrip and finger pinch - Amplitudes, conduction velocities and distal latencies | | **Primary outcomes:**   - NDS scores were similar between the two groups - Muscle strength, cutaneous sensation, cerebrospinal fluid protein, and nerve conduction were not significantly different between groups - Other measures of neurological function, such as touch-pressure of the hand (p=0.017), handgrip (p=0.046), and conduction velocity of motor fibers of the median nerve (p=0.029) showed improvement with prednisone compared to no treatment | 13 weeks | Dyck J.P., *et al. Ann Neurol.* 1982;11:136–141 |
| **RMC trial** | ISRCTN73774524 | Weekly 7.5 mg oral methotrexate (4 weeks) followed by 10 mg weekly (4 weeks) and 15 mg weekly (32 weeks) vs placebo | Pilot, multicenter, randomized, double-blind, controlled trial | | 59 | | **Primary endpoints:**   - >20% reduction in mean weekly dose in the last 4 weeks of the trial compared with the first 4 weeks   **Secondary endpoints:**   - Measured activity limitations and strength at mid-trial and final visits | | **Primary outcomes:**   - 52% taking methotrexate and 44% taking placebo had a >20% reduction in mean weekly dose of corticosteroids or IVIG (adjusted odds ratio 1·21, 95% CI 0·40–3·70)   **Secondary outcomes:**   - There were no clinically or statistically significant differences in secondary outcomes | 40 weeks | RMC Trial Group. *Lancet Neurol.* 2009 Feb;8(2):158–164 |
| **PREDICT** | 07779236^b^ | Daily oral prednisolone vs. monthly oral dexamethasone | Randomized, double blind controlled trial | | 40 | | **Primary endpoints:**   - Patients achieving remission at 12 months   **Secondary endpoints:**   - Quality of life factors | | **Primary outcomes:**   - 16 patients were in remission: 10 in the dexamethasone group and 6 in the prednisolone group   **Secondary outcomes:**   - AEs were minor and did not differ substantially between treatment groups | 39 weeks | van Schaik I.N., *et al*. *Lancet Neurol*. 2010;9(3):245–253 |
| **FcRn Blockers** | | | | | | | | | | | |
| **RCT** | [03861481](https://clinicaltrials.gov/ct2/show/study/NCT03861481?term=NCT03861481&rank=1) | Rozanolixizumab  10 mg/kg received 12 once-weekly or placebo infusions during an 11-week treatment period | Phase II, multicenter, randomized, subject blind, investigator blind, placebo controlled, parallel group trial | | 34 | | **Primary endpoints:**   - CFB to day 85 in the iRODS score - Efficacy | | **Primary outcomes:**   - Rozanolixizumab was well tolerated over medium-to-long-term weekly use, with an acceptable safety profile - No difference in CFB to day 85 in iRODS centile score between groups - 82% patients receiving rozanolixizumab experienced AEs - CIDP01 and CIDO02 were well tolerated over up to 614 days | 13 weeks | Querol L, *et al*. J *Neurol Neurosurg Psychiatry*. 2024;95(9):845–854 |
| **ADHERE** | 04281472 | Efgartigimod PH20 (1000 mg) for 12 weeks (Stage A) and 48 weeks (Stage B) every week vs placebo | Two-part, randomized, double-blinded, multicenter, placebo-controlled trial | | 322 | | **Primary endpoints:**   - Responders vs. placebo as defined by INCAT disability score   **Secondary endpoints:**   - iRODS, grip strength and AEs | | **Primary outcomes:**   - Stage A: 66.5% patients were treatment responders - Stage B: efgartigimod significantly reduced the risk of relapse vs. placebo (p=0.000039)   **Secondary outcomes:**   - iRODs and grip strength showed similar features as INCAT - Most AEs were mild/moderate | 60 weeks | Allen J.A., *et al*. *Lancet Neurol*. 2024 Oct;23(10):1013–1024 |
| **ARISE** | [05327114](https://clinicaltrials.gov/ct2/show/NCT05327114) | Nipocalimab  Stage A: Loading dose (dose 1) by IV on Day 1, followed by dose 2 by IV once every 2 weeks from week 2 to week 12  Stage B: will receive dose 2 IV every 2 weeks from day 1 to week 52 | Stage A: Phase II/III, randomized, double, placebo-controlled, multicenter clinical study  Stage B: Open-label extension study | | Target: 300 (recruiting) | | **Primary endpoints:**   - Efficacy and safety - INCAT disability score - MRC - Grip strength | | The study is currently enrolling patients, targeting approximately 300 patients, with primary study completion date anticipated in 2026 | Stage A: 12 weeks  Stage B: 52 weeks | Ford L. *et al*. *Neurol*. 2023; Protocol presented in AAN annual meeting 2024 |
| **Complement pathway inhibitors** | | | | | | | | | | | |
| **SAR445088** | 04658472 | Riliprubart (SAR445088), monoclonal antibody anti-C1s), across three groups of patients with CIDP | An open-label, phase II, proof of concept, non-randomized, multicenter study | | 90 | | **Primary endpoints:**   - The percentage of participants with a response compared to baseline in the SoC-refractory and SoC-naïve groups   **Secondary endpoints:**   - Safety, tolerability, immunogenicity, and efficacy of SAR445088 during 12 weeks | | The aim of this phase 2 study is to determine the efficacy, safety, and tolerability of SAR445088 in a broad spectrum of CIDP patient groups including patients treated with SoC therapies, patients refractory to SoC therapies, and patients who are naïve to SoC therapies | 24 weeks  Extension study: 52 weeks | Querol L, *et al*. *J Peripher Nerv Syst*. 2023;28:276–285 |
| **B-cell depletion treatments** | | | | | | | | | | | |
| **RECIPE** | 03864185 | Rituximab (anti-CD20 monoclonal antibody)  375 mg/m^2^ once weekly for 4 weeks  2:1 Rituximab to placebo | An open-label, randomized, double blind, placebo-controlled, multicenter, parallel-group, comparative study | | 15 | | **Primary endpoints:**   - INCAT disability scale   **Secondary endpoints:**   - I-RODS | | The results of the RECIPE study are expected to provide evidence for the positioning of rituximab as a pathogenesis-based therapeutic for refractory CIDP | 52 weeks | Shimizu S., *et al*. *JMIR Res Protoc*. 2020;9(4):e17117 |
| **Proof-of-concept rituximab study** | 05877040 | Rituximab  Dose: 1g in one day, followed by the same dose after two weeks | Open-label, prospective exploratory study | | 17 | | **Primary endpoints:**   - Determine the proportion of patients who showed improvement 6 months after rituximab therapy - Nerve conduction test | | **Primary outcomes:**   - 76.5% patients showed improvement at 6 months - 92.9% showed improvement at 12 months - Nerve conduction parameters improved by 20% in two nerves in 40% patients at 6 months and 53.9% at 12 months | 12 months | Doneddu P.E., *et al*. *J Neurol Neurosurg Psychiatry*. 2024;95(9):838–844 |
| **Rituximab versus placebo for chronic inflammatory demyelinating polyradiculoneuropathy: a randomized trial** | 06325943 | Rituximab (1 g on days 1, 15, and 180±7) or placebo  Both groups continued their regular immunoglobulin doses for six months post-intervention | Randomized, double-blind, placebo-controlled | | 37 | | **Primary endpoint:**   - Proportion of patients who worsened in any of the following measures at month 12, within six months after immunoglobulin discontinuation:   - Decrease of at least one point on the adjusted INCAT score   - Decrease of two points on the MRC sum score   - Or a decrease of four points on the RODS centile score   **Secondary endpoints:**   - Proportion of patients deteriorating at month 18 (within 12 months after immunoglobulin discontinuation) - Treatment cessation due to adverse events or voluntary reasons - Time until deterioration after immunoglobulin discontinuation | | **Primary outcomes:**   - A similar proportion of patients in rituximab (63%) and placebo (66%) groups worsened at month 12 (OR 0.86; 95% CI 0.22-3.32) - No significant difference was noted at month 18 (OR 0.62; 95% CI 0.14-2.70) or in the mean scores of each scale at months 6, 12, and 18 - Median time to worsening was 5 months for rituximab and 2 months for placebo (p=0.44) - Treatment suspended due to adverse events for one patient receiving rituximab | 12 months | Nobile-Orazio E. *et al. Brain.* 2024;awae400 |

40-MWT, 40-m-walking test; AMS, average muscle score; CFB, change from baseline; CI, confidence interval; CIDP, chronic inflammatory demyelinating polyneuropathy; EFNS/PNS, European Federation of Neurological Societies/Peripheral Nerve Society; EOS, End of study; fSCIG, facilitated subcutaneous immunoglobulin; FVC, forced vital capacity; IGIV-C, Immune Globulin IV (Human), 10% Caprylate/Chromatography Purified; INCAT, Inflammatory Neuropathy Cause and Treatment; I-RODS, Inflammatory-Rasch-built Overall Disability Scale; ISRCTN, International Standard Randomized Controlled Trial Number; IVIG, intravenous immunoglobulin; IVMP, intravenous methylprednisolone; MRC, Medical Research Council; N/A, not available; NCS, nerve conduction studies; NIS, Neurological Impairment Score; ODSS, Overall Disability Sum Score; OR, odds ratio; SoC, standard of care; SCIG, subcutaneous immunoglobulin.

## References

1. van Doorn PA, Vermeulen M, Brand A, Mulder PGH, Busch HFM. Intravenous Immunoglobulin Treatment in Patients with Chronic Inflammatory Demyelinating Polyneuropathy. *Archives of Neurology* (1991) 48(February).

2. Vermeulen M, van Doorn PA, Brand A, Strengers PF, Jennekens FG, Busch HF. Intravenous Immunoglobulin Treatment in Patients with Chronic Inflammatory Demyelinating Polyneuropathy: A Double Blind, Placebo Controlled Study. *J Neurol Neurosurg Psychiatry* (1993) 56(1):36-9. doi: 10.1136/jnnp.56.1.36.

3. Hahn AF, Bolton CF, Zochodne D, Feasby TE. Intravenous Immunoglobulin Treatment in Chronic Inflammatory Demyelinating Polyneuropathy. A Double-Blind, Placebo-Controlled, Cross-over Study. *Brain* (1996) 119 ( Pt 4):1067-77. doi: 10.1093/brain/119.4.1067.

4. Thompson N, Choudhary P, Hughes RA, Quinlivan RM. A Novel Trial Design to Study the Effect of Intravenous Immunoglobulin in Chronic Inflammatory Demyelinating Polyradiculoneuropathy. *J Neurol* (1996) 243(3):280-5. doi: 10.1007/BF00868527.

5. Mendell JR, Barohn RJ, Freimer ML, Kissel JT, King W, Nagaraja HN, et al. Randomized Controlled Trial of Ivig in Untreated Chronic Inflammatory Demyelinating Polyradiculoneuropathy. *Neurology* (2001) 56(4):445-9. doi: 10.1212/wnl.56.4.445.

6. Hughes RA, Donofrio P, Bril V, Dalakas MC, Deng C, Hanna K, et al. Intravenous Immune Globulin (10% Caprylate-Chromatography Purified) for the Treatment of Chronic Inflammatory Demyelinating Polyradiculoneuropathy (Ice Study): A Randomised Placebo-Controlled Trial. *Lancet Neurol* (2008) 7(2):136-44. doi: 10.1016/S1474-4422(07)70329-0.

7. Leger JM, De Bleecker JL, Sommer C, Robberecht W, Saarela M, Kamienowski J, et al. Efficacy and Safety of Privigen((R)) in Patients with Chronic Inflammatory Demyelinating Polyneuropathy: Results of a Prospective, Single-Arm, Open-Label Phase Iii Study (the Prima Study). *J Peripher Nerv Syst* (2013) 18(2):130-40. doi: 10.1111/jns5.12017.

8. Kuwabara S, Mori M, Misawa S, Suzuki M, Nishiyama K, Mutoh T, et al. Intravenous Immunoglobulin for Maintenance Treatment of Chronic Inflammatory Demyelinating Polyneuropathy: A Multicentre, Open-Label, 52-Week Phase Iii Trial. *J Neurol Neurosurg Psychiatry* (2017) 88(10):832-8. Epub 20170802. doi: 10.1136/jnnp-2017-316427.

9. Nobile-Orazio E, Pujol S, Kasiborski F, Ouaja R, Corte GD, Bonek R, et al. An International Multicenter Efficacy and Safety Study of Iqymune in Initial and Maintenance Treatment of Patients with Chronic Inflammatory Demyelinating Polyradiculoneuropathy: Prism Study. *J Peripher Nerv Syst* (2020) 25(4):356-65. Epub 20200831. doi: 10.1111/jns.12408.

10. Cornblath DR, van Doorn PA, Hartung HP, Merkies ISJ, Katzberg HD, Hinterberger D, et al. Randomized Trial of Three Ivig Doses for Treating Chronic Inflammatory Demyelinating Polyneuropathy. *Brain* (2022) 145(3):887-96. doi: 10.1093/brain/awab422.

11. Markvardsen LH, Debost JC, Harbo T, Sindrup SH, Andersen H, Christiansen I, et al. Subcutaneous Immunoglobulin in Responders to Intravenous Therapy with Chronic Inflammatory Demyelinating Polyradiculoneuropathy. *Eur J Neurol* (2013) 20(5):836–42. Epub 20130107. doi: 10.1111/ene.12080.

12. Markvardsen LH, Sindrup SH, Christiansen I, Olsen NK, Jakobsen J, Andersen H, et al. Subcutaneous Immunoglobulin as First-Line Therapy in Treatment-Naive Patients with Chronic Inflammatory Demyelinating Polyneuropathy: Randomized Controlled Trial Study. *Eur J Neurol* (2017) 24(2):412–8. Epub 20161221. doi: 10.1111/ene.13218.

13. van Schaik IN, Bril V, van Geloven N, Hartung HP, Lewis RA, Sobue G, et al. Subcutaneous Immunoglobulin for Maintenance Treatment in Chronic Inflammatory Demyelinating Polyneuropathy (Path): A Randomised, Double-Blind, Placebo-Controlled, Phase 3 Trial. *Lancet Neurol* (2018) 17(1):35-46. Epub 20171106. doi: 10.1016/S1474-4422(17)30378-2.

14. Bril V, Hadden RDM, Brannagan TH, 3rd, Bar M, Chroni E, Rejdak K, et al. Hyaluronidase-Facilitated Subcutaneous Immunoglobulin 10% as Maintenance Therapy for Chronic Inflammatory Demyelinating Polyradiculoneuropathy: The Advance-Cidp 1 Randomized Controlled Trial. *J Peripher Nerv Syst* (2023) 28(3):436-49. Epub 20230706. doi: 10.1111/jns.12573.

15. Hadden RDM, Andersen H, Bril V, Basta I, Rejdak K, Duff K, et al. Long-Term Safety and Tolerability of Hyaluronidase-Facilitated Subcutaneous Immunoglobulin 10% as Maintenance Therapy for Chronic Inflammatory Demyelinating Polyradiculoneuropathy: Results from the Advance-Cidp 3 Trial. *J Peripher Nerv Syst* (2024) 29(4):441–52. Epub 20241111. doi: 10.1111/jns.12672.

16. Dyck PJ, Daube J, O'Brien P, Pineda A, Low PA, Windebank AJ, et al. Plasma Exchange in Chronic Inflammatory Demyelinating Polyradiculoneuropathy. *N Engl J Med* (1986) 314(8):461-5. doi: 10.1056/NEJM198602203140801.

17. Hahn AF, Bolton CF, Pillay N, Chalk C, Benstead T, Bril V, et al. Plasma-Exchange Therapy in Chronic Inflammatory Demyelinating Polyneuropathy. A Double-Blind, Sham-Controlled, Cross-over Study. *Brain* (1996) 119 ( Pt 4):1055-66. doi: 10.1093/brain/119.4.1055.

18. Dyck PJ, O'Brien PC, Oviatt KF, Dinapoli RP, Daube JR, Bartleson JD, et al. Prednisone Improves Chronic Inflammatory Demyelinating Polyradiculoneuropathy More Than No Treatment. *Annals of Neurology* (1982) 11:136–41.

19. Group RT. Randomised Controlled Trial of Methotrexate for Chronic Inflammatory Demyelinating Polyradiculoneuropathy (Rmc Trial): A Pilot, Multicentre Study. *Lancet Neurol* (2009) 8(2):158-64. Epub 20090110. doi: 10.1016/S1474-4422(08)70299-0.

20. van Schaik IN, Eftimov F, van Doorn PA, Brusse E, van den Berg LH, van der Pol WL, et al. Pulsed High-Dose Dexamethasone Versus Standard Prednisolone Treatment for Chronic Inflammatory Demyelinating Polyradiculoneuropathy (Predict Study): A Double-Blind, Randomised, Controlled Trial. *Lancet Neurol* (2010) 9(3):245-53. Epub 20100202. doi: 10.1016/S1474-4422(10)70021-1.

21. Querol L, De Seze J, Dysgaard T, Levine T, Rao TH, Rivner M, et al. Efficacy, Safety and Tolerability of Rozanolixizumab in Patients with Chronic Inflammatory Demyelinating Polyradiculoneuropathy: A Randomised, Subject-Blind, Investigator-Blind, Placebo-Controlled, Phase 2a Trial and Open-Label Extension Study. *J Neurol Neurosurg Psychiatry* (2024) 95(9):845-54. Epub 20240816. doi: 10.1136/jnnp-2023-333112.

22. Querol L, Lewis RA, Hartung HP, Van Doorn PA, Wallstroem E, Luo X, et al. An Innovative Phase 2 Proof-of-Concept Trial Design to Evaluate Sar445088, a Monoclonal Antibody Targeting Complement C1s in Chronic Inflammatory Demyelinating Polyneuropathy. *J Peripher Nerv Syst* (2023) 28(2):276-85. Epub 20230531. doi: 10.1111/jns.12551.

23. Shimizu S, Iijima M, Fukami Y, Tamura N, Nakatochi M, Ando M, et al. Efficacy and Safety of Rituximab in Refractory Cidp with or without Igg4 Autoantibodies (Recipe): Protocol for a Double-Blind, Randomized, Placebo-Controlled Clinical Trial. *JMIR Res Protoc* (2020) 9(4):e17117. Epub 20200401. doi: 10.2196/17117.

24. Doneddu PE, Cocito D, Fazio R, Benedetti L, Peci E, Liberatore G, et al. Prospective Open-Label Trial with Rituximab in Patients with Chronic Inflammatory Demyelinating Polyradiculoneuropathy Not Responding to Conventional Immune Therapies. *J Neurol Neurosurg Psychiatry* (2024) 95(9):838-44. Epub 20240816. doi: 10.1136/jnnp-2023-332844.

25. Nobile-Orazio E, Cocito D, Manganelli F, Fazio R, Lauria Pinter G, Benedetti L, et al. Rituximab Versus Placebo for Chronic Inflammatory Demyelinating Polyradiculoneuropathy: A Randomized Trial. *Brain* (2024). Epub 20241210. doi: 10.1093/brain/awae400.

26. Allen JA, Lin J, Basta I, Dysgaard T, Eggers C, Guptill JT, et al. Safety, Tolerability, and Efficacy of Subcutaneous Efgartigimod in Patients with Chronic Inflammatory Demyelinating Polyradiculoneuropathy (Adhere): A Multicentre, Randomised-Withdrawal, Double-Blind, Placebo-Controlled, Phase 2 Trial. *Lancet Neurol* (2024) 23(10):1013-24. doi: 10.1016/S1474-4422(24)00309-0.
